# The role of ammonia in chronic liver disease

**DOI:** 10.1097/HC9.0000000000000993

**Published:** 2026-07-15

**Authors:** Annarein J.C. Kerbert, Maria Pilar Ballester, Christopher F. Rose, Rajiv Jalan

**Affiliations:** 1 Department of Gastroenterology and Hepatology, Leiden University Medical Center, Leiden, The Netherlands; 2 Digestive Disease Department, Hospital Clínico Universitario de Valencia, Spain; 3 INCLIVA Biomedical Research Institute, Valencia, Spain; 4 Hepato-Neuro Laboratory, CRCHUM, Université de Montréal, Montreal, Quebec, Canada; 5 Institute for Liver and Disease Health, University College London, London, UK

**Keywords:** biomarker, cirrhosis, neurotoxicity, risk stratification, therapeutic target

## Abstract

Hepatic encephalopathy (HE) is a major neuropsychiatric complication of chronic liver disease that contributes substantially to morbidity, mortality, and healthcare burden. Ammonia, a neurotoxic metabolite, plays a central role in its pathogenesis. Although traditionally considered a byproduct of liver failure, growing evidence supports a more direct and causal role for ammonia, linking it not only to the clinical features of HE but also to key processes involved in disease progression, including liver injury, inflammation, immune dysfunction, muscle wasting, and impaired cellular energetics. Beyond its mechanistic implications, renewed interest has focused on ammonia as a clinically useful biomarker. Advances in risk stratification, dynamic testing, and longitudinal monitoring are improving the ability to use ammonia levels to identify patients at high risk of HE, predict clinical outcomes, and assess response to therapy. At the same time, emerging ammonia-lowering therapies are reshaping the treatment landscape. Whether these therapies may modify disease trajectory, beyond reversing HE, is an interesting hypothesis requiring validation in future studies. This review summarises current knowledge on ammonia metabolism, its role in the pathophysiology of HE and chronic liver disease, and its evolving clinical utility. By integrating molecular mechanisms with advances in measurement and treatment, we propose a reframing of ammonia as a central driver of liver–brain axis dysfunction and a meaningful target for precision-based therapeutic strategies.

## Graphical abstract

**Figure:**
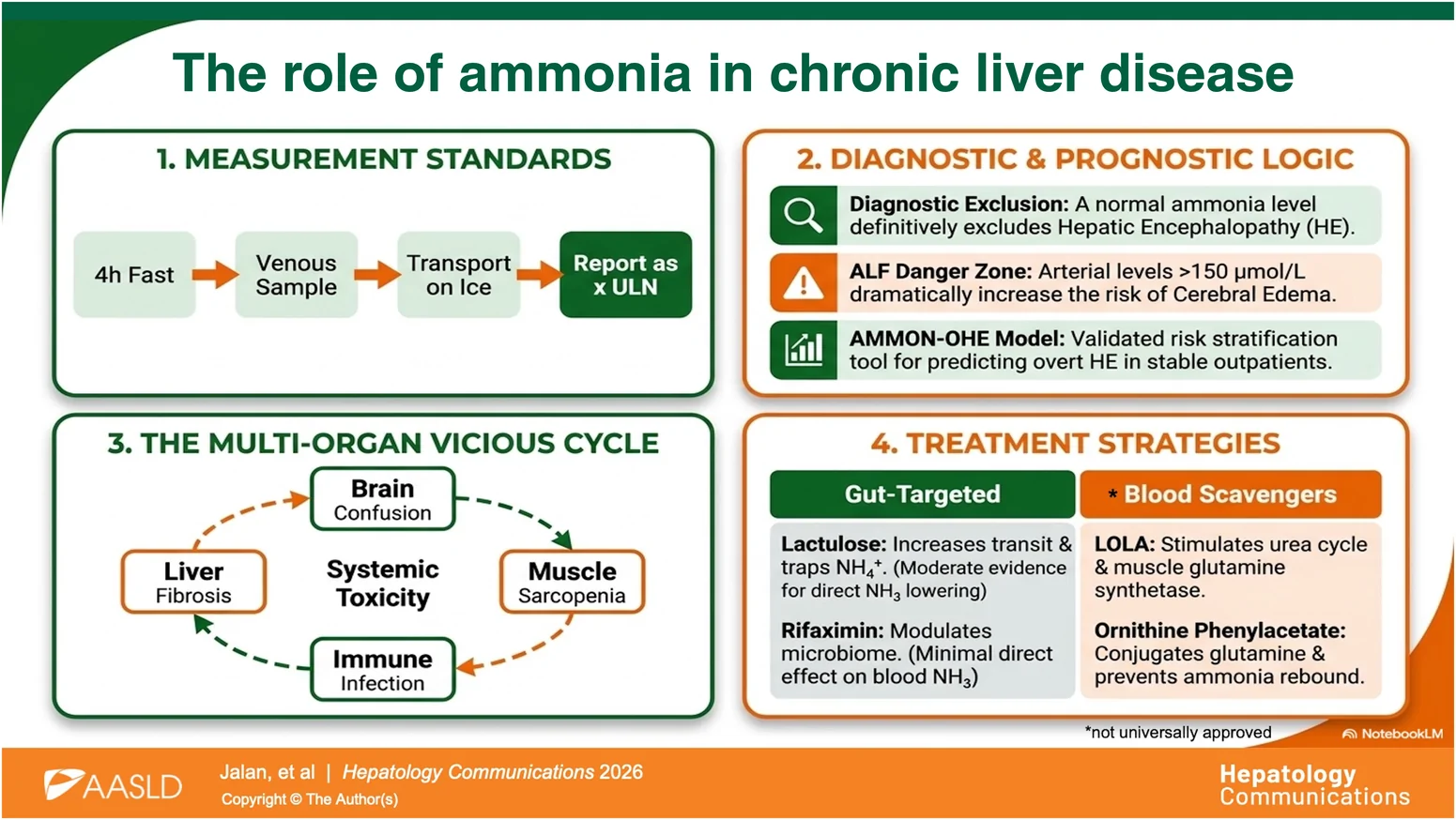

## INTRODUCTION

Hepatic encephalopathy (HE) remains one of the most debilitating complications of chronic liver disease, encompassing neuropsychiatric abnormalities ranging from subtle cognitive impairment to coma.^1^^,^^2^ It affects up to 40% of patients with cirrhosis during the disease course.^3^ Despite decades of investigation, HE continues to impose a substantial burden on patients, caregivers, and healthcare systems, reflecting both its high prevalence and its association with recurrent hospitalizations, impaired quality of life, and excess mortality.^4^^–^^7^ Central to HE pathogenesis is ammonia, a small nitrogenous molecule. Long recognized as a neurotoxin, ammonia has re-emerged as a key mechanistic driver of liver–brain dysfunction and a candidate biomarker of disease activity and risk.^8^

Under physiological conditions, ammonia is detoxified by the liver and muscles through urea synthesis and glutamine formation. Chronic liver disease disrupts this balance. Hepatocellular dysfunction, portosystemic shunting, sarcopenia, and gut microbiome alterations impair ammonia clearance, leading to hyperammonemia.^9^ While elevated ammonia levels have historically been viewed as a biochemical epiphenomenon of liver failure, accumulating evidence supports a more causal role, linking ammonia not only to the clinical manifestations of HE but also to broader processes that influence disease progression, including progressive liver injury, inflammation, immune dysfunction, muscle wasting, and impaired cellular energetics.^10^^–^^12^

The relationship between ammonia and HE is complex. Single plasma ammonia measurements correlate imperfectly with neurological status, contributing to skepticism regarding clinical utility.^13^ However, this limitation likely reflects methodological and conceptual challenges rather than biological irrelevance. Ammonia exists in multiple compartments, fluctuates dynamically, and interacts synergistically with systemic inflammation, infection, and organ failure, which are key features of advanced cirrhosis and acute decompensation.^14^^,^^15^ Emerging data suggest that ammonia is best understood not as a standalone marker but as part of a pathophysiological network in which it amplifies neurotoxicity by inducing astrocyte swelling, oxidative stress, mitochondrial dysfunction, and neuroinflammation.^2^

At the cellular level, ammonia exerts deleterious effects across multiple organs. In the brain, it disrupts astrocyte metabolism, neurotransmission, and cerebral energy homeostasis.^16^ In skeletal muscle, ammonia drives catabolism and suppresses protein synthesis, reducing an important extrahepatic detoxification site and worsening hyperammonemia.^11^ In the liver, dysregulated ammonia metabolism can lead to fibrogenesis and hepatocyte death.^10^^,^^17^ Systemically, ammonia modulates immune cell function and inflammatory signaling, reinforcing the concept that HE is a disorder of integrated organ failure rather than solely brain dysfunction.^12^^,^^14^^,^^18^

Against this mechanistic backdrop, renewed interest has focused on ammonia as a measurable and actionable biomarker. Advances in risk stratification, dynamic testing, and longitudinal assessments have begun to redefine how ammonia levels may identify patients at risk of HE, predict outcomes, and monitor therapeutic response. In parallel, novel ammonia-lowering strategies are reshaping the therapeutic landscape. This perspective is reflected in the first Delphi Consensus publication on ammonia by the International Society for Hepatic Encephalopathy and Nitrogen Metabolism (ISHEN), summarized in Box 1.^19^

Box 1 Recommendations on ammonia measurement. Principal recommendations of the International Delphi consensus on the role of ammonia measurement in patients with cirrhosis by the International Society for Hepatic Encephalopathy and Nitrogen metabolism (ISHEN).^19^

**Table:** 

*Methodological issues in ammonia measurement*
- Ammonia should be measured with a minimum of a 4-hour fasting period to reduce the variability in ammonia levels caused by recent dietary intake.
- Venous ammonia sampling is recommended as it is safe and simple to access and correlates well with arterial ammonia sampling.
- Plasma ammonia sampling may provide greater reproducibility than serum or whole blood.
- A laboratory-based assay is recommended as it is more accurate than point-of-care tests.
- Ammonia should be reported both as absolute values and as a multiple of the upper limit of normal of a reference standard to provide more reproducible results between laboratories.
- Blood should be sampled, transported on ice (4 °C), centrifuged within 15 minutes, and plasma ammonia should be measured within 2 hours.
*Role of measuring ammonia in the outpatient setting*
- Ammonia concentration may be useful to improve the risk prediction for the occurrence of OHE.
- Ammonia concentration may be useful to improve the risk prediction for non-HE liver-related decompensation and death.
- An ammonia concentration of >1.4 times the upper limit of normal can be useful to stratify the risk of hospitalization with liver-related decompensation and death.
- Elevated blood ammonia is not required for the diagnosis of MHE (minimal hepatic encephalopathy).
- Elevated blood ammonia is not required to initiate primary prophylaxis of OHE.
- Measuring blood ammonia before TIPS may be useful to improve the risk prediction for post-TIPS OHE.
- Measuring blood ammonia within the first week after TIPS may improve the risk prediction for the occurrence of OHE.
*Role of measuring ammonia in the inpatient setting*
- Measuring blood ammonia may help differentiate OHE from other causes of encephalopathy.
- Blood ammonia concentration may not be useful to differentiate the severity of OHE.
- Measuring blood ammonia may be useful to predict the risk of mortality.
- Measuring blood ammonia may be useful to predict the risk of developing OHE.
- Blood ammonia measurements should be repeated within 2–7 days after initiation of OHE therapy to predict resolution of OHE in patients without adequate response.

This article synthesizes current understanding of ammonia metabolism, its mechanistic role in HE and chronic liver disease, and its evolving utility as a biomarker. By bridging molecular insights with clinical measurement and therapeutic innovation, we aim to reframe ammonia not as an imprecise laboratory value, but as a central, biologically meaningful driver of liver–brain dysfunction and a target for precision intervention.

## MECHANISMS OF AMMONIA TOXICITY

### Neurotoxicity

Ammonia is a well-established neurotoxin and central driver of HE pathophysiology. During hyperammonemia, ammonia enters the brain via passive diffusion, specific transporters, and potassium channels, leading to elevated cerebral ammonia levels.^20^ Multiple brain cell types are affected. The associated evidence, mechanisms, and effects are described below and summarized in Figure 1. It is important to note that most of the discussed mechanistic studies have been performed in in-vitro models, limiting their generalizability in patients.

**FIGURE 1:**
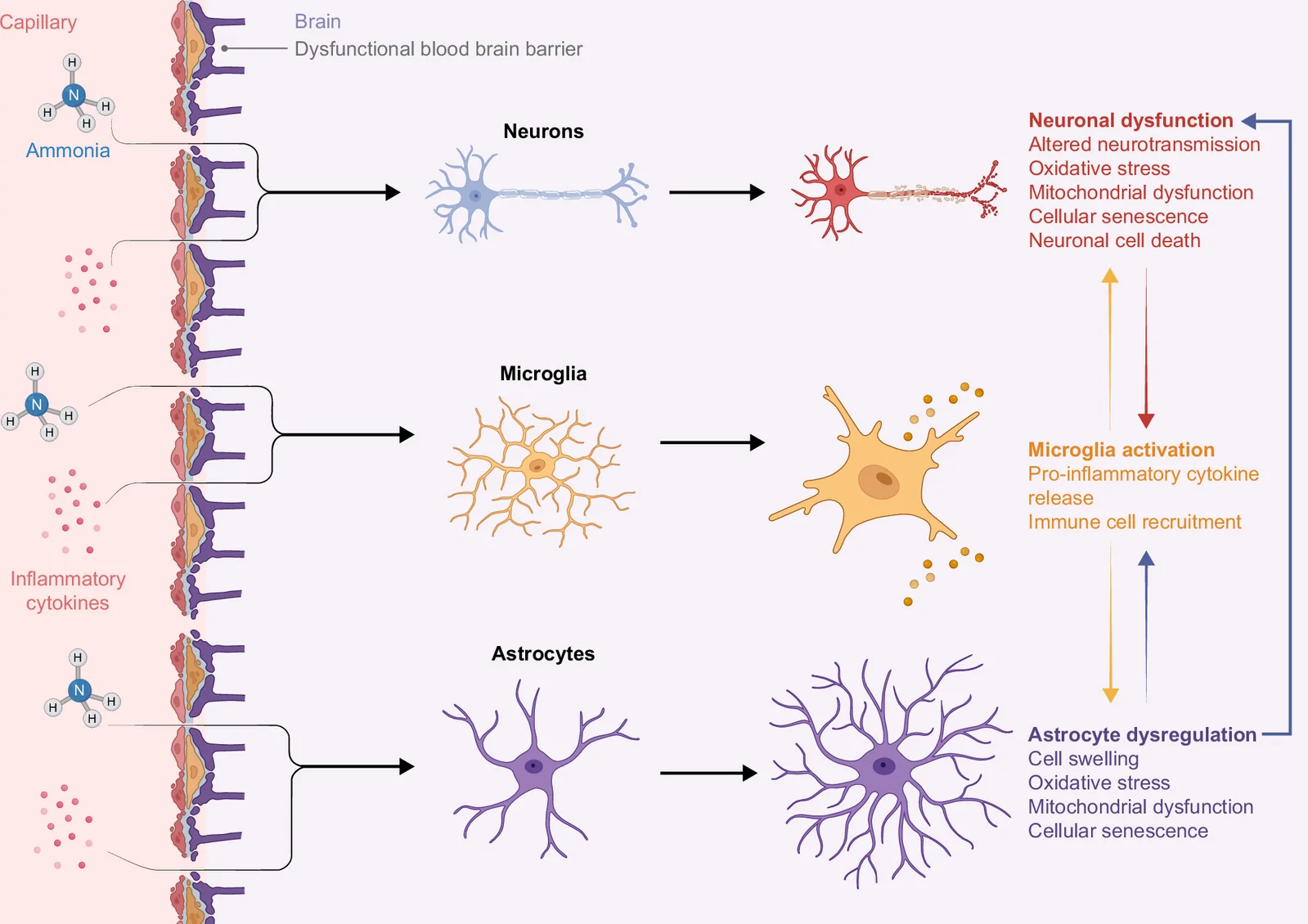
The neurotoxic effects of ammonia. Ammonia and systemic pro-inflammatory molecules synergistically exert their neurotoxic effects on several brain cell types. Astrocyte dysregulation is a consistent feature of HE, with ammonia shifting astrocyte function from supportive to detrimental. Excess glutamine generated via glutamine synthetase accumulates within astrocytes and enters mitochondria, where it is converted to glutamate by glutaminase and then deaminated to α-ketoglutarate to fuel the tricarboxylic acid cycle. Both reactions generate additional ammonia, promoting reactive oxygen species production through mitochondrial pH alterations and inhibition of key enzymatic processes, including components of the respiratory chain. These processes increase mitochondrial membrane permeability, leading to swelling, fragmentation, and bioenergetic failure that may drive astrocyte senescence. Neurons possess limited intrinsic antioxidant defenses and rely on astrocytic support for amino acid and glutathione shuttling, making them highly susceptible to oxidative stress during chronic hyperammonemia. This may result in cellular senescence and neuronal death. Microglia, the resident innate immune cells, are activated by ammonia and peripheral inflammation, leading to the release of pro-inflammatory cytokines and recruitment of immune cells that contribute to neuroinflammation and subsequent astrocytic and neuronal injury. Abbreviation: HE, hepatic encephalopathy.

#### Astrocytes

Astrocytes are traditionally considered the primary cellular targets of ammonia toxicity because they uniquely detoxify ammonia through glutamine synthetase (GS). The pathogenesis has traditionally been attributed to astrocyte swelling and low-grade cerebral edema caused by osmotic imbalance secondary to astrocytic glutamine accumulation. However, this paradigm has been increasingly questioned.^2^^,^^21^ In contrast to acute liver failure (ALF), only minimal changes in brain water content have been observed in patients with cirrhosis-associated HE, and its contribution to neurological dysfunction remains controversial.^21^^–^^24^

Nevertheless, astrocyte dysregulation is a consistent feature of HE. Numerous in vitro, ex vivo, and animal studies have demonstrated astrocytic glutamine accumulation during hyperammonemia.^21^ Glutamine enters mitochondria, where conversion to glutamate and α-ketoglutarate generates additional ammonia and reactive oxygen species (ROS), impairing respiratory chain activity and mitochondrial integrity.^25^^,^^26^

The resulting oxidative stress promotes astrocyte swelling, protein modification, transcriptional dysregulation, and altered inflammatory signaling.^27^^–^^30^ Studies in ammonia-treated rat astrocytes further suggest that ammonia-induced oxidative stress may drive astrocyte senescence via glutamine-dependent ROS production, p53 activation, and cell cycle inhibition, potentially contributing to irreversible cognitive impairment.^31^^,^^32^ Increased expression of senescence-associated genes has also been demonstrated in post-mortem brains of cirrhotic patients with HE.^31^

#### Neurons

Neurons may represent a critical vulnerability in HE as their limited antioxidant defense mechanisms and dependence on astrocytic metabolic support render them susceptible to oxidative stress during chronic hyperammonemia.^33^ Ex vivo studies have demonstrated increased ROS production, lipid peroxidation, and neuronal death in bile duct-ligated (BDL) rats, effects attenuated by ornithine phenylacetate (OP).^34^ Repeated hyperammonemic episodes in BDL rats have been found to further induce hippocampal oxidative stress and neuronal loss.^35^

Recent evidence suggests that cellular senescence may preferentially affect neurons rather than astrocytes. In BDL mice, senescence markers were predominantly detected in hippocampal neurons.^36^ These findings highlight the need for mechanistic studies addressing neuronal vulnerability and neuron-astrocyte interactions during hyperammonemia.

#### Microglia and neuroinflammation

Systemic and neuroinflammation are central components of HE pathophysiology. Peripheral inflammation resulting from liver dysfunction and gut dysbiosis contributes to central inflammatory responses, as demonstrated by correlations between circulating inflammatory markers and cerebral pro-inflammatory gene expression in both cirrhotic patients with/without HE (post-mortem brain samples) and cirrhotic rat models.^29^^,^^37^^–^^39^ Microglia, the resident innate immune cells of the central nervous system, play a key role by releasing pro-inflammatory cytokines and facilitating immune cell recruitment. Increased expression of the microglial activation marker Iba1 has been demonstrated in post-mortem HE brains and BDL rats.^40^^–^^43^ Transcriptomic analyses further demonstrate upregulated expression of inflammatory cytokines in microglia from BDL rat brains.^27^

Ammonia itself appears capable of modulating microglial activation. In vitro and rodent studies show that ammonia-induced activation is associated with mitochondrial ROS production and inflammasome activation.^41^^–^^46^ However, liver-specific GS knockout and acute ammonia intoxication models demonstrate minimal microglial activation, suggesting that hyperammonemia alone may be insufficient and additional inflammatory signals are required.^40^

Other brain cell types may also contribute to neuroinflammation. Astrocytes from BDL rats demonstrate inflammatory transcriptomic signatures, while hyperammonemic diet models show elevated tumor necrosis factor expression in Purkinje cells and hippocampal neurons.^27^^,^^43^^,^^47^^,^^48^ Although the functional consequences of microglia activation in HE remain incompletely defined, evidence from animal models implicates a role in neurodegeneration, synaptic loss, and altered GABAergic and glutamatergic neurotransmission.^27^^,^^49^^,^^50^

#### Oxygen homeostasis and the glymphatic system

Hyperammonemia also disrupts broader cerebral homeostasis. A recent study demonstrated that chronic hyperammonemia in BDL rats reduces cortical oxygenation, likely through impaired systemic and cerebral perfusion.^51^ Disturbances in cerebral oxygen delivery and utilization have been associated with cognitive and neurological dysfunction in chronic hyperammonemia. In addition, the brain in cirrhosis appears more vulnerable to hypotensive insults, which may result in neuronal cell death.^52^ Pharmacological lowering of ammonia in a BDL model has been shown to prevent hypotension-induced neuronal loss, highlighting the role of ammonia in increasing cerebral vulnerability.^53^ The mechanisms by which ammonia alters cerebral perfusion remain incompletely understood, but increased GABAergic tone and subsequent decreased neuronal activity, energy consumption, and cerebral oxygen demand have been proposed to play a role.^54^ These changes may additionally impair the glymphatic system, the brain’s waste-clearance pathway, promoting accumulation of metabolic waste products and worsening cerebral dysfunction.^55^ The clinical significance of impaired oxygen homeostasis and glymphatic dysfunction in HE remains uncertain. Longitudinal neuroimaging and neuropsychological studies may provide insight into whether these mechanisms contribute to the cumulative cognitive deficits observed after repeated HE episodes.

### Toxicity to extra-cerebral organs and systems—evidence and open questions

Beyond neurotoxicity, hyperammonemia exerts detrimental systemic effects that may explain why circulating ammonia predicts poor outcomes in both stable and acutely decompensated cirrhosis.^56^ Accumulating studies demonstrate that ammonia contributes to sarcopenia, immune dysfunction, and progressive liver injury (Figure 2). This section aims to outline the associated evidence and open questions.

**FIGURE 2:**
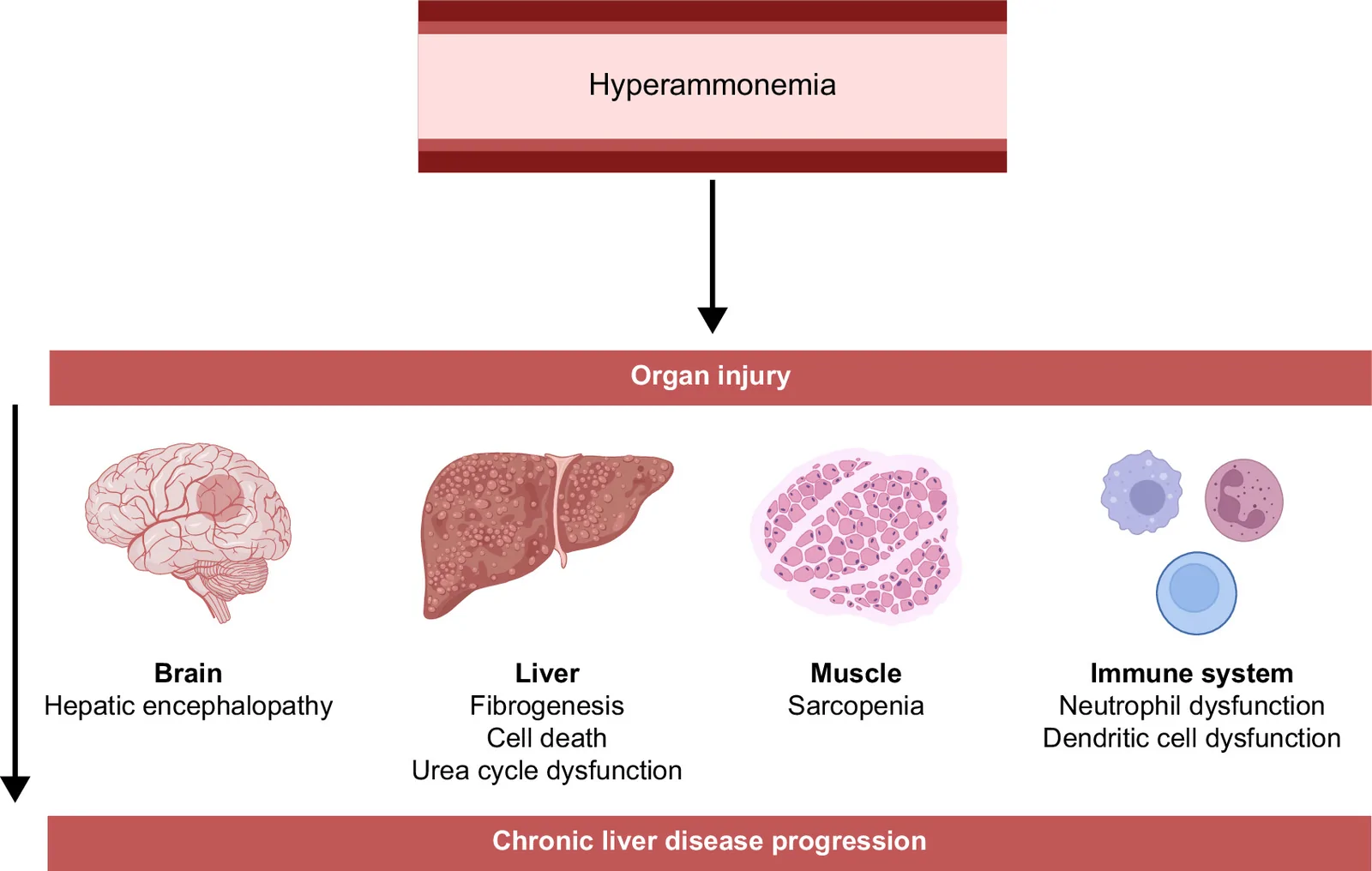
Impact of hyperammonemia on peripheral tissues. Beyond its neurotoxic effects, the detrimental extra-cerebral consequences of hyperammonemia have received increasing attention over the past decades. These systemic effects may explain why circulating ammonia levels are a strong prognostic marker in both clinically stable and acutely decompensated patients, independent of the presence of HE. Specifically, hyperammonemia has been shown to induce injury to the liver (fibrogenesis, cell death, urea cycle dysfunction), skeletal muscle (sarcopenia), and immune cells (neutrophil and dendritic cell dysfunction). Abbreviation: HE, hepatic encephalopathy.

#### Sarcopenia

Sarcopenia affects 40%–70% of patients with cirrhosis and strongly predicts mortality.^57^ Hyperammonemia contributes directly to muscle wasting, while sarcopenia further worsens hyperammonemia because skeletal muscle serves as an alternative ammonia detoxification site. Ammonia induces myostatin expression through nuclear factor κB signaling and suppresses mammalian target of rapamycin complex 1 (mTORC1) activity, impairing muscle growth and protein synthesis. It also induces oxidative stress, mitochondrial dysfunction, and cellular senescence in muscle cells.^58^ Both preclinical and clinical studies demonstrate that ammonia-lowering therapies preserve muscle mass and function.^59^^–^^61^

#### Immune function

A few studies suggest that hyperammonemia impairs innate immune function, particularly affecting neutrophils and dendritic cells. In vitro exposure of isolated neutrophils from healthy individuals to ammonia, as well as studies in rats fed with an ammoniagenic diet, revealed neutrophil swelling, reduced phagocytic capacity, and increased spontaneous oxidative burst.^12^ Similarly, in cirrhotic patients with hyperammonemia induced by an amino acid solution, neutrophil phagocytosis was significantly impaired compared with controls. Mechanistic studies implicate activation of the p38 mitogen-activated protein kinase pathway in ammonia-induced neutrophil dysfunction.^12^

Dendritic cells isolated from patients with cirrhosis exhibit reduced antigen uptake relative to healthy controls. Experimental studies indicate that ammonia induces mitochondrial injury in dendritic cells, characterized by mitochondrial swelling, increased ROS production, and loss of membrane potential. In mouse models of hyperammonemia, dendritic cell phagocytosis and antigen-specific immune responses, including responses to ovalbumen vaccination, are markedly attenuated.^18^ Together, these findings suggest that hyperammonemia contributes to the immunocompromised state observed in cirrhosis, although a direct association between ammonia levels and infection risk has yet to be established.

#### Hepatotoxicity

Emerging preclinical studies suggest that the liver itself is vulnerable to ammonia-induced injury. In isolated hepatic stellate cells, ammonia has been shown to promote its activation and pro-fibrogenic signaling.^17^ In BDL rats, treatment with OP reduced plasma ammonia levels, along with portal pressure and a reduction in hepatic stellate cell activation markers. More recent evidence from mouse models of isolated hyperammonemia demonstrated ammonia-induced fibrogenesis and necroptotic hepatocyte death.^10^ This injury was found to be associated with urea cycle dysfunction, likely establishing a vicious cycle that further exacerbates hyperammonemia. Pharmacological inhibition of TLR4 or RIPK1 disrupted this cycle by reducing ammonia levels and preventing hepatocyte death.^10^ To what extent hyperammonemia meaningfully contributes to liver injury progression in cirrhotic patients, where numerous other activating stimuli are present, remains to be investigated.

#### Clinical implications of the underlying mechanisms

The mechanisms described above remain incompletely understood and are largely derived from preclinical studies. In patients, ammonia neurotoxicity is likely to be more heterogeneous, influenced by liver disease severity, systemic inflammation, age, comorbidities, frailty, brain reserve, and precipitating factors. Systemic inflammation appears particularly important, as it increases cerebral susceptibility to ammonia toxicity while hyperammonemia may further amplify inflammation.^14^^,^^15^^,^^48^^,^^62^ Brain reserve may also explain variability in HE manifestations.^63^ Greater cognitive reserve may delay HE onset and reduce severity, whereas lower reserve increases vulnerability, especially in covert HE. Cognitive stimulation, physical activity, and adequate nutrition may strengthen resilience.^63^

HE has additionally been linked to neurodegenerative processes. Transcriptomic analyses have revealed similarities between HE and Alzheimer’s disease, particularly in pathways related to neuroprotection and neurodifferentiation.^64^ These similarities may partly arise from gut dysbiosis, highlighting the importance of the gut–brain axis in maintaining brain health.^65^ Ammonia itself may promote neurodegeneration through neuronal senescence and cell death, potentially explaining persistent cognitive dysfunction after liver transplantation.^66^^–^^68^ Ammonia has also been directly implicated in the pathogenesis of Alzheimer’s disease. Increased brain ammonia levels have been reported in Alzheimer’s disease and may promote amyloidogenesis in astrocytes.^69^^,^^70^ Moreover, metabolomic studies in patients with Alzheimer’s disease demonstrate disturbances in ammonia metabolism.^71^

Together, these observations suggest that inflammation, brain frailty, and neurodegeneration significantly modulate both acute and long-term effects of ammonia toxicity. Greater attention to these concepts may improve understanding of HE and identify more personalized therapeutic targets.

## EVIDENCE FOR ASSOCIATION OF AMMONIA WITH HE AND ROLE AS A BIOMARKER

As described above, ammonia occupies a central and causal position in the pathogenesis of HE, functioning not merely as an associated metabolite but as a mechanistic driver of neurotoxicity and a clinically informative biomarker.^16^^,^^72^ Although the relationship between hyperammonemia and HE has long been recognized, historical skepticism regarding ammonia measurement has limited its clinical adoption. Recent studies have provided greater clarity regarding its usefulness as a biomarker.^1^^,^^73^ One challenge in comparing ammonia levels across hospitals and studies is variability in defining a “normal” value. To harmonize reporting across the world, ammonia levels have been proposed to be expressed relative to the upper limit of normal (ULN) of the local laboratory. Using this approach, emerging evidence supports ammonia as a biologically meaningful and clinically actionable biomarker across chronic liver disease. Understanding its strengths and limitations is essential for optimizing its role in hepatology practice. Figure 3 summarizes the clinical role of ammonia measurement in risk stratification, diagnosis, and treatment response, along with the associated available evidence.

**FIGURE 3:**
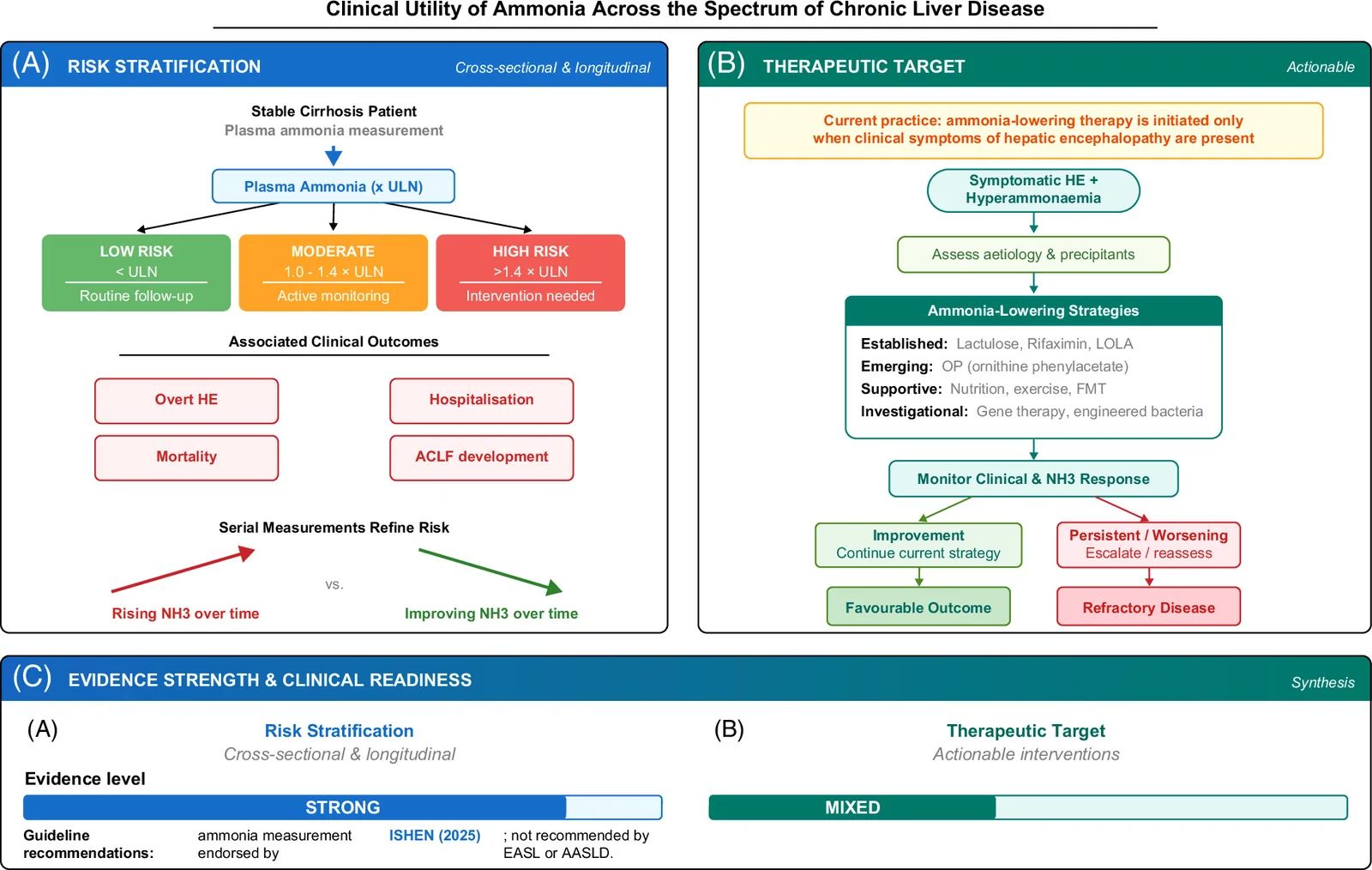
Clinical utility of ammonia across the spectrum of chronic liver disease. Proposed algorithms for ammonia measurement in stable cirrhosis (A) and in the setting of HE (B). The associated available evidence is reflected in section (C). Abbreviations: AASLD, American Association for the Study of Liver Diseases; ACLF, acute-on-chronic liver failure; FMT, fecal microbiota transplantation; HE, hepatic encephalopathy; ISHEN, International Society for Hepatic Encephalopathy and Nitrogen Metabolism; LOLA, L-ornithine L-aspartate; OP, ornithine phenylacetate; ULN, upper limit of normal.

### Strengths of ammonia as a biomarker

#### Diagnostic relevance in acute encephalopathy in cirrhosis

In patients with liver disease presenting with delirium or encephalopathy, a normal ammonia level challenges the diagnosis of HE and mandates evaluation for alternative causes.^1^ The EASL and ISHEN Consensus identify the presence of hyperammonemia necessary for the diagnosis of HE.^19^ However, in a retrospective French cohort of 530 patients hospitalized for acute decompensation, encephalopathy without hyperammonemia accounted for 31% of cases. These patients had higher rates of ascites and antibiotic use, whereas patients with hyperammonemia (69%) experienced more recurrent encephalopathy and worse 1-year transplant-free survival.^74^ Whether these non-hyperammonemic episodes should be considered classical HE remains a matter of debate.^75^ Likewise, the proposed term “hyperammonemic HE” remains controversial and is not aligned with current definitions requiring hyperammonemia for HE diagnosis. Taken together, ammonia measurement provides clinically relevant risk stratification, enabling more accurate diagnosis, targeted treatment, and surveillance.

#### Correlation with disease severity and outcomes in acute decompensation and ACLF

Large-scale studies in patients with acute decompensation and acute-on-chronic liver failure (ACLF) demonstrate that ammonia correlates with chronic liver disease severity,^76^^,^^77^ portal hypertension and systemic inflammation,^77^^,^^78^ organ failure burden, and mortality.^79^^,^^80^ In a prospective study of 498 patients with acute decompensation or ACLF, plasma ammonia independently predicted 28-day mortality. Admission ammonia ≥79.5 μmol/L identified patients at higher risk of liver, coagulation, kidney, and respiratory failure.^81^ A meta-analysis of 81 studies confirmed elevated ammonia levels in cirrhosis and HE, particularly overt HE (OHE), and demonstrated associations with creatinine, albumin, sodium, and inflammatory markers.^82^ Together, these findings position ammonia as an integrative marker of multisystem dysfunction.

#### Risk stratification and prediction of OHE in stable cirrhosis

Ammonia is among the strongest predictors of incident OHE in stable outpatients with cirrhosis.^76^^,^^83^ In a multicentre European study of 754 patients, ammonia independently predicted hospitalization and mortality and outperformed traditional severity scores.^76^ These findings were replicated in cohorts exceeding 600 patients.^77^^,^^83^ Importantly, the prognostic value of ammonia was independent of hepatic dysfunction, systemic inflammation, and portal hypertension severity, supporting a direct toxic role.^77^

The AMMON-OHE model, incorporating ammonia, sex, diabetes, albumin, and creatinine, demonstrated excellent discrimination and outperformed the psychometric hepatic encephalopathy score (PHES) and critical flicker frequency (CFF) in predicting OHE.^84^ In multivariable analysis, ammonia emerged as an independent predictor of overt HE, whereas PHES and CFF did not.^84^

In patients undergoing transjugular intrahepatic portosystemic shunt (TIPS), baseline ammonia consistently predicted OHE in prospective German studies.^85^^,^^86^ A retrospective analysis of 278 TIPS recipients confirmed that the AMMON-OHE model outperformed liver function scores, with age, diabetes, albumin, and ammonia identified as independent pre-TIPS predictors.^87^

#### Ammonia as a biomarker of treatment response

Ammonia reduction underpins the mechanism of action of most established HE therapies, yet ammonia measurement remains underutilized. In a phase 2b study of OP for OHE,^88^ HE improvement was directly associated with ammonia reduction.^89^ No treatment effect was observed in patients without hyperammonemia, suggesting that ammonia may serve as a companion biomarker for selecting patients for ammonia-lowering therapy. Similarly, in the cohort of 498 patients with acute decompensation mentioned before, failure of ammonia improvement by day 5 was associated with high 28-day mortality (70.6%), highlighting its value as a dynamic response biomarker.^81^ A preliminary study using sodium phenylbutyrate in critically ill patients with OHE and ammonia >100 μmol/L similarly demonstrated that ammonia lowering was associated with neurological improvement and improved outcomes.^90^ Whole-body ammonia flux studies demonstrate reduced ammonia clearance and increased production in cirrhosis. Combination therapy with lactulose and rifaximin reduced ammonia production by 20%, whereas phenylbutyrate increased ammonia clearance by 11%.^91^ These findings validate ammonia as a quantitative marker of treatment efficacy.

#### Diagnostic and prognostic value in ALF

Hyperammonemia is a hallmark metabolic disturbance in ALF and plays a central role in HE and cerebral edema. Abrupt loss of hepatic urea cycle function results in rapid systemic and cerebral ammonia accumulation. Ammonia crosses the blood–brain barrier and is metabolized in astrocytes to glutamine, promoting astrocytic swelling, oxidative stress, and mitochondrial dysfunction, mechanisms believed to underlie the development of cerebral edema and intracranial hypertension.^92^ Several clinical studies demonstrate strong associations between ammonia levels and neurological injury severity in ALF. Elevated ammonia correlates with HE grade and the risk of intracranial hypertension. Clemmesen et al^93^ showed a strong association between high arterial and cerebral ammonia concentrations and cerebral edema leading to herniation. A more recent study showed that arterial ammonia >100 μmol/L independently predicted severe HE and intracranial hypertension.^94^ In a large US cohort, admission ammonia was significantly higher in patients with grade 3–4 HE than in those with lower grades.^95^ Ammonia concentrations >150 μmol/L have been associated with markedly increased risk of cerebral edema and brain herniation.^96^ These observations have important clinical implications because cerebral edema remains one of the leading causes of death in ALF, particularly in patients with higher HE grades.^95^ Unlike chronic liver disease, adaptive tolerance is absent, rendering ammonia a critical biomarker in this setting.^97^

Ammonia also has prognostic value in ALF. Higher admission ammonia levels are associated with increased mortality and worse neurological outcomes.^98^ In a recent multicenter ALF cohort, higher ammonia independently predicted lower transplant-free survival after adjustment for sex, etiology, and lactate.^99^ In addition to predicting mortality, ammonia concentrations can help identify patients at increased risk of complications requiring aggressive management or early consideration for liver transplantation. Ammonia thresholds >150–200 μmol/L are often used to guide decisions regarding intensive monitoring, including intracranial pressure monitoring in selected patients.^92^ Incorporating ammonia into prognostic models improves prediction of severe encephalopathy and neurological complications in ALF.^94^^,^^99^

### Limitations of ammonia as a biomarker

#### Imperfect correlation with HE grades

Correlation between ammonia and HE severity is imperfect. While many studies demonstrate positive associations,^81^^,^^100^^–^^102^ others report weaker relationships,^34^^,^^103^ likely reflecting interindividual differences in cerebral vulnerability.^82^^,^^104^ As discussed, factors including brain reserve, inflammation, and compartmentalized ammonia exposure contribute to this variability.^105^

#### Elevated ammonia levels without HE

Some patients exhibit elevated ammonia levels despite normal mental status. This likely reflects the multifactorial nature of neurocognitive dysfunction rather than biomarker inadequacy. Ammonia levels are also influenced by diet, muscle mass, exercise, medications, and nutritional state.^106^^–^^109^ Nevertheless, hyperammonemia predicts poor outcome in any clinical situation, whether in the outpatient department, as an inpatient, prior to TIPS, or in ALF.

#### Pre-analytical variability and measurement challenges

Pre-analytical variability remains a major technical challenge, with sample handling, transport time, temperature, and assay methodology significantly influencing results.^110^^–^^113^ A UK national audit demonstrated wide variability in ammonia measurement practices and artefactual increases caused by delayed processing.^112^^,^^113^ These limitations are potentially remediable through standardization. Reporting ammonia relative to ULN may improve interpretation across centers and studies.

#### Clinical implications

Ammonia is a mechanistically grounded, prognostically powerful, relatively inexpensive, and therapeutically relevant biomarker available in most hospitals. While its diagnostic specificity for HE severity is limited when interpreted in isolation, its greatest value lies in diagnosis, risk stratification, prediction of OHE, prognostication, and monitoring treatment response. Emerging evidence supports a shift from historical skepticism toward structured and standardized ammonia measurement in cirrhosis. When interpreted in a clinical context, ammonia provides biological insight not captured by conventional liver scores or psychometric testing and should be reinstated as a core component of precision care in chronic liver disease. A summary of the recent ISHEN Delphi Consensus recommendations is outlined in Box 1.^19^

## AMMONIA-TARGETED THERAPIES: CURRENT AND FUTURE

Despite the established role of ammonia in the pathophysiology of HE and its potential use as a biomarker, there is a relative paucity of therapeutic targeting of ammonia as the primary therapy of HE. Treatment of acute episodes is largely aimed at addressing the precipitating events, and specific therapies in most parts of the world are limited to the use of lactulose for all stages of HE and rifaximin for the prevention of recurrence of OHE in patients with a previous episode.^1^ Neither of these drugs is a typical “ammonia-removing” agent, but rather decreases gut ammonia absorption.

### Non-ammonia-targeted approaches

#### Lactulose

This non-absorbable disaccharide is recommended as first-line treatment for HE.^1^ Even though lactulose was never FDA-approved for the treatment of HE, it was initially prescribed to treat constipation (a common precipitating event of HE). Its primary mode of action is via increasing the intestinal transit, thereby reducing systemic ammonia absorption. In addition, lactulose has been shown to alter the gut microbiota, potentially promoting non-urease-producing bacteria and suppressing urease-producing proteolytic bacteria to subsequently reduce bacterial breakdown of nitrogenous substrates into ammonia.^114^ Moreover, lactulose has been shown to acidify the bowel milieu, which increases the ionic form of ammonia (NH4^+^), which is less permeable compared with its gaseous form (NH_3_), across the intestinal membrane. This ‘trapping’ of NH4^+^ has been suggested to lead to increased fecal nitrogen excretion. However, consistent reduction in ammonia levels with the use of lactulose has not been demonstrated in controlled clinical trials.^89^ A Cochrane review demonstrated that the treatment of lactulose had moderate quality evidence of benefit for HE, and evidence for lowering plasma ammonia was of very low quality.^115^ Lactulose is very affordable, but compliance remains a challenge since treatment is associated with adverse events, including diarrhea, nausea, bloating, and flatulence.

#### Rifaximin

Rifaximin, a semi-synthetic non-aminoglycoside, acts against gram-positive, gram-negative, aerobic, and anaerobic enteric bacteria. This is the first and only FDA-approved treatment for the prevention of recurrence of overt HE based on a phase 3 clinical trial.^116^ Since then, several trials aiming to explore the use of this drug in decompensated cirrhosis patients have been negative.^117^^–^^119^ Very recently, the top-line results of the 2 phase 3 trials in about 1000 patients (RED-C-3131/NCT05071716 and RED-C-3132/NCT05297448) have been negative as well, arguing whether rifaximin is indeed a disease-modifying agent in patients with cirrhosis. It is important to note that the impact of rifaximin on blood ammonia levels is minimal,^120^ especially since rifaximin is primarily prescribed as an add-on therapy to lactulose. Importantly, the STOP-HE study, a phase 2b trial evaluating the safety and efficacy of OP, showed that the standard of care (lactulose ± rifaximin) did not lower ammonia levels in patients with OHE.^89^ Minimal effect of the combination on ammonia levels was observed in another randomized trial of oral OP versus rifaximin plus lactulose.^121^

Even though it is minimally absorbed, rifaximin has been shown to influence the function of the gut microbiome as opposed to relative bacterial abundance.^122^ More recently, 3 studies have suggested that the use of rifaximin is likely to be associated with increased risk of antimicrobial resistance, suggesting that antibiotic stewardship for this drug may well be required.^123^^–^^125^

#### Other approaches

Approaches to treat HE, such as probiotics, supplementation of groups of bacteria, and fecal microbiome transplantation targeting the gut microbiome, are in development, but do not directly target ammonia metabolism.^8^

### Ammonia-targeting strategies

#### Interorgan metabolism as the basis for targeting ammonia

Most of the ammonia generation arises from the gut, which is then easily absorbed into the portal venous system. The liver’s “first pass” allows for the efficient detoxification of ammonia by converting it into urea in the periportal hepatocytes by the urea cycle, which is subsequently excreted by the kidneys.^126^ In addition, perivenous hepatocytes use glutamine synthetase (GS) to convert any remaining ammonia into glutamine. However, ammonia is an important substrate, as well as an important product, for over 20 different biochemical reactions. An additional prominent enzymatic reaction involves ammonia phosphate-activated glutaminase (GLS). Whereas GS catalyzes the amination of glutamate to glutamine, which removes a molecule of ammonia, the reciprocal reaction involves GLS, which deaminates glutamine to glutamate, liberating a molecule of ammonia. In addition to the liver, ammonia homeostasis involves the participation of multiple organs that house GS or GLS. In order to reduce blood ammonia levels, increasing ureagenesis via stimulation of the urea cycle is an obvious therapeutic target. However, with hepatocyte death or severe injury, targeting the liver remains less appealing. Reducing ammonia absorption from the gut is a key therapeutic strategy, as is increasing ammonia removal via stimulation of GS.^2^^,^^9^ This section discusses the current and future therapeutic strategies for ammonia lowering (Figure 4).

**FIGURE 4:**
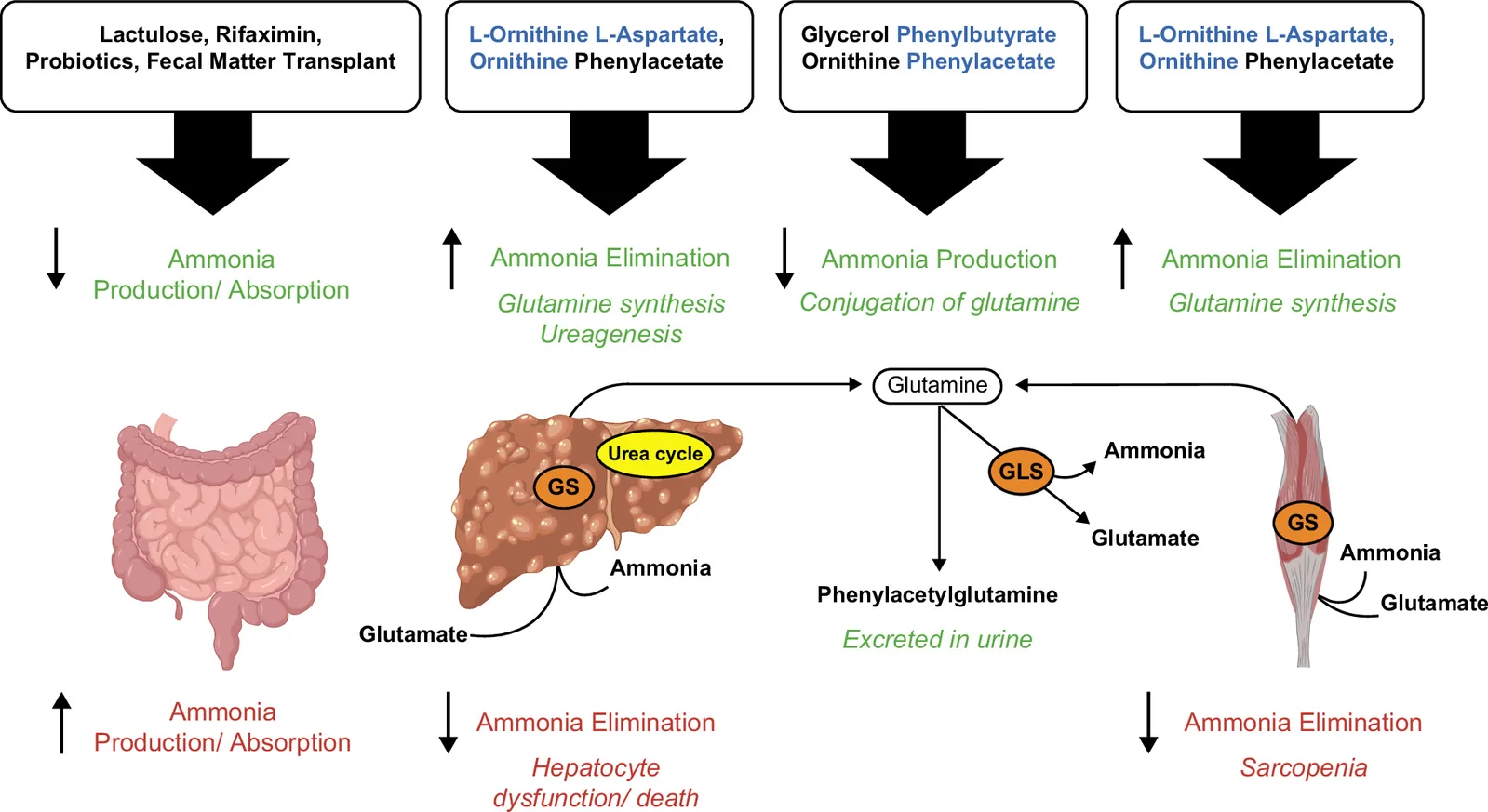
Therapeutic strategies for the treatment of HE. The gut is a major source of ammonia generation via ammoniagenic bacteria. Therefore, altering the microbiome or increasing fecal ammonia excretion is an approach to reduce ammonia absorption into the systemic circulation. An alternative strategy is to increase ammonia removal by targeting the urea cycle within the residual hepatocytes or stimulating glutamine synthetase in the muscle with L-ornithine L-aspartate or ornithine phenylacetate. Phenylacetate (glycerol phenylbutyrate) conjugates with glutamine to avoid glutamine-derived ammonia generation via glutaminase. Ornithine phenylacetate combines the removal of ammonia via the stimulation of GS with ornithine as well as the conjugation of glutamine with phenylacetate to prevent an ammonia rebound. Abbreviations: GLS, glutaminase; GS, glutamine synthetase; HE, hepatic encephalopathy.

### Drugs in clinical use that have an effect on ammonia production or excretion

#### L-ornithine L-aspartate

L-ornithine L-aspartate (LOLA) is a stable salt consisting of 2 amino acids that increases urea synthesis in residual hepatocytes as well as GS synthesis in muscle.^127^ Multiple studies have demonstrated that LOLA reduces plasma ammonia in patients with cirrhosis, especially after intravenous administration. Although not approved by the federal agencies in many countries, LOLA has been shown to be beneficial for the treatment of HE. However, the positive effect of the oral formulation of LOLA has been debated in various meta-analyses, resulting from differences in trial inclusion and exclusion criteria.^128^

### Drugs in development

#### Ornithine phenylacetate

OP is a stable salt composed of L-ornithine and phenylacetate, where L-ornithine increases GS in muscle and phenylacetate chelates ammonia-derived glutamine to form phenylacetylglutamine. The latter product is not metabolized by GLS as glutamine is, therefore preventing an ammonia rebound effect. The drug has been tested and validated in about 8 early-stage clinical trials, providing evidence of its safety, robust effect on ammonia lowering, and success in patients with both cirrhosis and ALF with or without renal dysfunction.^89^^,^^129^ OP completed a phase 2b clinical trial in patients with OHE.^88^ The study failed to meet its primary endpoint, most likely because of the erroneous inclusion of 30 patients (out of 231 randomized patients) who did not have elevated ammonia levels. However, after excluding these patients in a post hoc analysis,^89^ the study showed statistical significance in time to resolution of HE. The exploratory findings of this pharmacodynamic analysis will need to be validated in future trials. The study also demonstrated that improvement in HE was associated with a decrease in plasma ammonia.^89^ In further developments, an oral form of this drug was studied in a phase 2a trial and showed excellent pharmacokinetics and ability to reduce ammonia concentration in patients with decompensated cirrhosis.^121^ A phase 2b/3 trial for the prevention of occurrence and recurrence of OHE using the oral formulation is being planned.

#### Glycerol phenylbutyrate

Glycerol phenylbutyrate (GPB) is a typical glutamine scavenger, which acts by removing glutamine from the circulation and preventing ammonia generation. This drug has been shown to have an excellent safety profile with the ability to prevent the occurrence and recurrence of encephalopathy in patients with urea cycle enzyme disorders. A phase 2 study in patients with cirrhosis provided the proof of concept that it can significantly reduce plasma ammonia concentrations and HE events.^130^ For reasons that are not clear, this agent has not been developed further in the field of chronic liver disease.

### Future perspectives

The evolving understanding of ammonia as both a mechanistic driver and a prognostically powerful biomarker creates an opportunity to fundamentally redesign therapeutic development in HE. Patient selection in future trials of ammonia-lowering interventions should involve HE patients with confirmed hyperammonemia instead of enrollment based on a clinical HE diagnosis only. Emerging data indicate that ammonia should function as a *companion biomarker*, with ammonia-lowering therapies developed specifically in patients with confirmed hyperammonemia and stratified using validated ammonia-based risk tools such as the AMMON-OHE model. Treating patients using ammonia-lowering drugs without biochemical evidence of ammonia excess risks diluting therapeutic signals and repeating historical failures.^88^^,^^89^ Having said this, the level of evidence, although emerging, is still sketchy, and further prospective studies are needed to define the role of using ammonia levels as a therapeutic target in clinical practice.

Technological advances should also prioritize improved ammonia measurement, including standardized pre-analytical protocols, reporting relative to institutional ULNs, and development of point-of-care or dynamic monitoring tools. Ammonia is an excellent biomarker but only when measured correctly and interpreted in a clinical context, accounting for inflammation, renal function, muscle mass, nutritional state, and disease trajectory. Integration of ammonia with inflammatory markers, sarcopenia metrics, and organ failure scores will enable multidimensional risk profiling and support personalized treatment pathways.

At the therapeutic level, next-generation ammonia-targeted strategies, including metabolic scavengers, microbiome modulation, and extracorporeal clearance platforms, offer the prospect of sustained ammonia control. Although it has been established that ammonia-lowering strategies reverse HE episodes, the hypothesis discussed in this review that (preventive) ammonia-lowering may impact disease progression in cirrhosis needs validation in adequately designed trials. These studies should evaluate whether chronic ammonia reduction can modify systemic manifestations of cirrhosis, including sarcopenia, immune dysfunction, and progression to acute-on-chronic liver failure, rather than focusing exclusively on neurocognitive endpoints.

Ultimately, embedding ammonia into structured care algorithms to guide early intervention, monitor response, and inform TIPS selection represents a key step toward precision hepatology.

## CONCLUSIONS

Ammonia has re-emerged as a central determinant of HE and chronic liver disease progression. Far from being a nonspecific laboratory value, ammonia is a mechanistically grounded, prognostically robust, and therapeutically actionable biomarker integrating liver dysfunction with systemic and cerebral injury. When measured rigorously and interpreted in context, ammonia provides clinical insight not captured by conventional liver scores or psychometric testing. The future of HE management lies in aligning mechanism, measurement, and medicine. By repositioning ammonia as both a disease driver and guide to intervention, the field may move beyond symptomatic management toward true disease modification.
